# Graphene-Enabled Tunable Phase Gradient Metasurface for Broadband Dispersion Manipulation of Terahertz Wave

**DOI:** 10.3390/mi14112006

**Published:** 2023-10-28

**Authors:** Yin Zhang, Yijun Feng, Junming Zhao

**Affiliations:** 1 School of Information Engineering, Nanjing University of Finance and Economics, Nanjing 210023, China; 370820542@163.com; 2 Department of Electronic Engineering, School of Electronic Science and Engineering, Nanjing University, Nanjing 210023, China; yjfeng@nju.edu.cn

**Keywords:** graphene metasurface, broadband dispersion, phase gradient, abnormal reflection, beam focusing

## Abstract

With the increasing demand for the miniaturization and flexibility of optical devices, graphene-based metasurfaces have emerged as a promising ideal design platform for realizing planar and tunable electromagnetic or optical devices. In this paper, we propose a tunable metasurface with low-dispersion phase gradient characteristics that is composed of an array of double-layer graphene ribbons sandwiched with a thin insulating layer and a polymer substrate layer with a gold ground plane. As two typical proof-of-concept examples, metasurfaces act as a planar prism and a planar lens, respectively, and the corresponding performances of tunable broadband dispersion are demonstrated through full-wave simulation experiments. By changing the Fermi level of each graphene ribbon individually to introduce abrupt phase shifts along the metasurface, the broadband continuous dispersion effect of abnormal reflection and beam focusing is achieved within a terahertz (THz) frequency region from 3.0 THz to 4.0 THz, and the dispersion results can be freely regulated by reconfiguring the sequence of Fermi levels via the bias voltage. The presented graphene metasurface provides an avenue for the dispersion manipulation of a broadband terahertz wave and may have great prospects in the fields of optics, imaging, and wireless communication.

## 1. Introduction

Dispersion is the phenomenon of the phase velocity of a wave depending on its frequency and always plays a significant role in ultrashort optics, substance composition detection, optical instruments, communications technology, etc. [1,2,3]. But the traditional dispersive devices, such as prisms, are usually large in volume or thickness with respect to the wavelength of light, making miniaturized applications difficult to achieve. Metasurfaces, as planar artificial structural materials, have gained increasing attention in recent years due to their excellent ability to mold the flow of light in a desired manner [4,5,6]. In sharp contrast to traditional optical components, which depend on the accumulation of a spatial phase along the optical path, a metasurface relies on the abrupt phase change at the interface produced by subwavelength resonators for the deep manipulation of electromagnetic (EM) waves [7,8,9,10,11]. This has greatly promoted the development of the miniaturization of EM components. Many optical phenomena or optical devices can be realized based on the various designed ultrathin optical components [12,13,14,15,16,17,18], and the operating frequencies of these devices can cover optical and microwave wavelength ranges [19].

Recently, many devices based on dispersive metasurfaces have emerged for beamforming control relying on the frequency of incident waves. The variation in excitation frequency can be used to achieve the spatial scanning of reflected or transmitted beams within a certain range of angles [20,21] and control the focal length of dispersion-engineered metalenses [22,23]. Some metasurfaces have also shown different functions in different frequency bands based on their dispersion characteristics, such as reflection and transmission, diffuse and specular reflection, and dual-beam and quad-beam in two discrete wavebands [24,25,26]. These frequency-dependent control approaches are important in that they provide significantly valuable reference ideas for the manipulation of beamforming based on metasurfaces. But the available dispersion bandwidth is limited, thus restricting the variable range of beam direction or focal length and the flexibility of manipulation. Another advantage of metasurfaces is the enabling of dynamic or tunable manipulation of EM waves through external stimuli such as electrical bias, mechanical deformation, and thermal effects [27,28,29,30]. The intervention of external stimuli will greatly enhance the tunability of a metasurface and the flexibility of dispersion manipulation.

Electrical control is a practical and convenient means of achieving tunable functions. Graphene, a two-dimensional honeycomb lattice composed of densely packed carbon atoms, presents itself as a favorable material for metasurface integration to attain such tunability [31,32,33]. This is due to graphene’s sheet conductivity that can be dynamically modulated over a broad range of frequencies using external electrostatic biasing or chemical doping to adjust the Fermi level [34,35,36]. Graphene metasurfaces have been utilized in developing tunable optical devices with varied functions [37,38,39,40,41]. This has created prospects for the advancement of graphene-based metasurfaces in aspects of size, weight, and power. By arranging a graphene sheet in a particular pattern on top of a dielectric layer, a reflective or transmissive metasurface can be fabricated. In particular, graphene-enabled tunable phase gradient metasurfaces have achieved dynamic reconfigurability and abundant functions in the infrared and THz frequency ranges [42,43,44,45,46]. For example, dynamic beam steering and focusing can be realized by altering the phase of individual meta-atoms driven by graphene [42]. Beam steering has also been achieved by changing the graphene sheet resistance and combining it with the coding principle [43]. A kind of terahertz spatial varifocal metamirror, whose focus can be flexibly adjusted, is proposed based on a metal–graphene hybrid structure in Ref. [44]. The device proposed in Ref. [45] can be used to design optimal optical components capable of dynamic wavefront shaping, including perfect absorption, arbitrary multi-focus, and self-healing. By tuning the Fermi energy of graphene, dynamic reconfiguration among single- and multi-beam deflections, diffusive scattering, and vortex beams can be effectively achieved for a tunable phase gradient metasurface [46]. This enhances the active properties of a metasurface and enables dynamic spatial light modulation over an ultrathin surface. The response of EM waves can then be dynamically controlled in the desired manner [47]. Based on a graphene metasurface, it is possible to flexibly switch the dispersion results as demanded and significantly extend the spatial range of beam manipulation by combining dispersion and tunability.

However, designing a metasurface with broadband continuous dispersion characteristics that can be dynamically tuned, providing more freedom to manipulate the THz beam, is still a key technological challenge. In this work, we propose a graphene-enabled tunable metasurface with low-dispersion phase gradient characteristics over a broadband frequency range in the THz regime. The metasurface consists of an array of double-layer graphene ribbons isolated with a thin insulating layer on a gold mirror with a polymer gap substrate in between. The presented device can not only achieve wideband continuous dispersion effects, but also regulate the dispersion results expediently and dynamically. As two typical proof-of-concept examples, tunable dispersion of anomalous reflection and beam focusing are given to illustrate the proposed scheme. Full-wave simulation experiments reveal that the abnormal reflection and beam focusing achieved by a metasurface working at different states show a dispersion effect over a wide THz band from 3.0 THz to 4.0 THz, and the abnormal reflection angle and focal distance can be dynamically manipulated by rearranging the Fermi level of graphene ribbons through different voltage biasing. Thanks to the broadband dispersion characteristics and dynamic tunability of the metasurface, the variable spatial range of beam reflection and beam focusing is further extended, enhancing the flexibility of the device in potential applications. The presented graphene metasurface offers broadband dispersion with tunable operation for a variety of potential applications, such as optics, imaging, and wireless communication, and this may provide an effective solution for the design of new compact planar optical devices.

## 2. Structure and Methods

### 2.1. Structure Design

**Figure 1:**
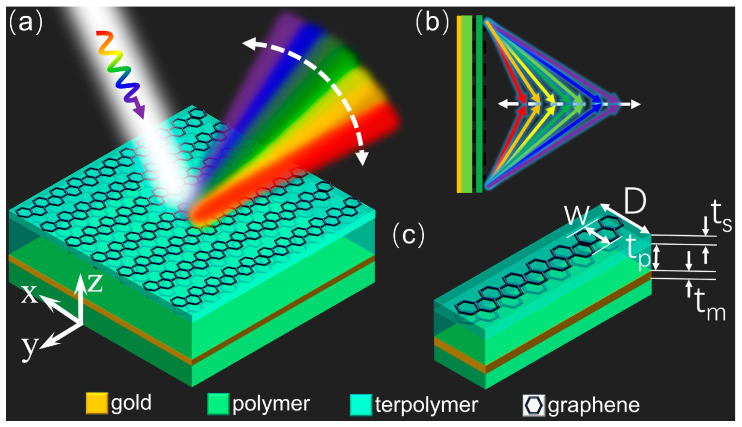
(**a**) Schematic diagram of the proposed metasurface and tunable dispersion effect of the abnormal reflection. (**b**) Schematic diagram of the tunable dispersion effect of beam focusing. (**c**) Geometry view of a unit cell.

Figure 1 depicts both the schematic of the designed metasurface and the details of its unit cell, which is a multilayer composite structure with a subwavelength thickness of approximately one-fifth of the excitation wavelength. As shown in Figure 1a, the metasurface is composed of a double layer of graphene ribbons on the top, a metal ground plane at the bottom, and a flexible dielectric substrate in between. The metal ground plane is made of gold film (a lossy metal) with a conductivity of *σ* = 4.56 × 10^7^ S/m, and the plane behaves as a perfectly reflecting layer in the THz regime. Due to its low absorption and almost dispersionless refractive index (approximately 1.53) across the THz band, TOPAS polymer is favored as the middle dielectric substrate material for broadband THz devices [48]. It is necessary to incorporate at least two layers of capacitively coupled graphene separated with a thin dielectric spacer to enable tuning of the Fermi level of graphene via external electrostatic gating [37]. Each capacitor structure in the unit cell array of the metasurface presented here consists of double layers of graphene ribbons sandwiched with a thin insulating layer, forming a gated element. Each sheet in the graphene ribbon pairs serves as a gate electrode, which allows for dynamic control of the desired wideband dispersion effects achieved by the metasurface. This is achieved by applying a direct current voltage that shifts the Fermi level in both graphene layers in opposing directions. An approximate closed-form expression to relate the Fermi level and the bias voltage is given by Ref. [35]:

(1) $$E_{F}\approx \hbar \nu_{f}\sqrt{\frac{\pi \varepsilon_{r}\varepsilon_{0}V_{g}}{et_{s}}}$$

where *E*_F_ is the Fermi level; *ε*_0_ and *ε_r_* are the permittivities of the vacuum and the dielectric spacer between the double layers of graphene, respectively; *t_s_* is the thickness of the dielectric spacer; and *e* and *ν_f_* are the electron charge and the Fermi velocity (1.1 × 10^6^ m/s in graphene), respectively. *V_g_* is the bias voltage applied to dynamically change the Fermi level of graphene. Generally, the Fermi level can be adjusted extensively, typically ranging from −1.0 eV to 1.0 eV. To ensure the safety of the electrical bias, a polyvinylidene fluoride (PVDF)-based terpolymer, with a high permittivity of 65 and a dielectric breakdown strength of 500 V/μm or more [49], is employed as the material of the thin insulating spacer between double layers of graphene.

Then, using the graphene-enabled tunable metasurface, the tunable broadband dispersion effect of abnormal reflection and beam focusing can be achieved, as schematically shown in Figure 1a,b, and the dispersion results can be freely regulated for broadband THz waves. All of the geometrical parameters of the unit cell, as shown in Figure 1c, are set after performance optimization through full-wave simulation for an EM field. The width *w* of the graphene ribbon and the period *D* of the unit cell are 10 μm and 12 μm, respectively. The thickness *t_s_* of the insulating spacer between graphene sheets and the *t_p_* of the polymer substrate are 0.3 μm and 17 μm, respectively. The ground plane has a thickness *t_m_* of 0.2 μm, which is much larger than the typical skin depth in the working frequency regime and ensures that the device works in reflection mode.

### 2.2. Simulation Methods

To demonstrate the prospective dispersion formation and manipulation ability of the graphene-enabled tunable metasurface, full-wave simulation experiments were performed to investigate the EM responses of the metasurface using the commercial software program CST Microwave Studio 2016. In the EM simulation, the terpolymer insulation layer, polymer substrate, and gold ground plane are modeled as a standard cube model by using their corresponding materials (pre-defined or imported from the material library) and geometrical parameters. The graphene monolayer can be modeled as a two-dimensional conductive sheet with a complex-valued surface conductivity, *σ_s_* (*ω*, *E*_F_, *Γ*, *T*), connected to the Fermi level *E*_F_ via the bias voltage. Here, *ω* represents the radian frequency, *Γ* (*Γ* = 1/2*τ*, where *τ* is the electron–phonon relaxation time) represents the phenomenological scattering rate, and *T* is the environmental temperature. Throughout this research, it is assumed that the relaxation time *τ* = 0.5 ps and the environmental temperature *T* = 300 K [50]. The sheet conductivity of graphene can be derived using the well-established Kubo formula and is described through interband and intraband contributions as follows [36]:

(2) $$\sigma_{S}=\sigma_{intra}(\omega ,E_{F},\Gamma ,T)+\sigma_{inter}(\omega ,E_{F},\Gamma ,T)$$

(3) $$\sigma_{intra}(\omega ,E_{F},\Gamma ,T)=-j\frac{e^{2}k_{B}T}{\pi \hbar^{2}(\omega -j2\Gamma )}(\frac{E_{F}}{k_{B}T}+2\ln(e^{-E_{F}/k_{B}T}+1))$$

(4) $$\sigma_{\mathrm{inter}}(\omega ,E_{F},\Gamma ,T)=\frac{-je^{2}}{4\pi \hbar }\ln(\frac{2\left|E_{F}\right|-(\omega -j2\Gamma )\hbar }{2\left|E_{F}\right|+(\omega -j2\Gamma )\hbar })$$

where *e*, *ħ*, and *k_B_* are universal constants representing the electron charge, Planck’s constant, and Boltzmann’s constant, respectively.

The unit cell has a symmetric structure in relation to both the *x-* and *y*-axes. Therefore, the perfect electrical conductor and perfect magnetic conductor boundary conditions are applied in the *x-* and *y*-directions, respectively. The open boundary condition is introduced in the *z*-direction in free space, which is tantamount to employing periodic boundary conditions for a normally incident wave. In the simulation of far-field characteristics, the metasurface, which has a finite size, is subjected to open boundary conditions along the *x*-, *y*-, and *z*-directions in free space. It is assumed that a THz wave beam polarized in the *x*-direction impinges normally on the metasurface along the *z*-direction. Due to the obstruction of the bottom gold ground plane, only the reflection phase and amplitude spectra need to be calculated and recorded in the simulation for a unit cell. To correspondingly obtain the far-field characteristics of the metasurface, plane wave excitation with *x*-polarization is employed. The time-domain solver carries out the specific calculation. The amplitude and phase spectra of the unit cell will be calculated, along with the far-field radar cross-section (RCS) and electric field distribution of the metasurface. Once computed, these results will be recorded in the navigation tree’s results folder. By analyzing the simulation results gathered by the preset field monitors at the expected frequencies, one can evaluate the performances of the reflected wave.

### 2.3. Potential Fabrication Process

**Figure 2:**
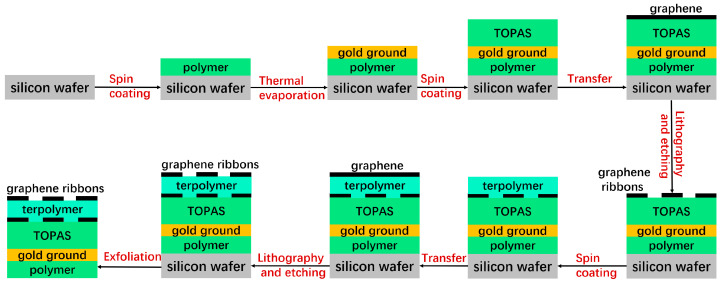
Potential fabrication process of the proposed metasurface.

Due to the advancements in transferring graphene films onto specific substrates and the rapid progress in preparing large-area, high-quality graphene on metal oxide surfaces, recent experimental demonstrations of graphene metasurfaces provide valuable references for the potential fabrication process of the proposed structure. These experiments suggest that the metasurface can be practically implemented [50,51,52]. Consequently, the suggested device structure can be produced by adopting a comparable technique, and the demanded designs of graphene/insulator inventories can be generated by recapitulating the growth and transfer process. As shown in Figure 2, the gold ground is formed via thermal evaporation on a silicon substrate that has been preliminarily spin-coated with a thin polymer layer. Next, a TOPAS substrate with a predetermined thickness is fabricated through spin-coating and baking on the groundwork. The graphene double layers, separated by a PVDF-based terpolymer buffer layer, are fabricated on the TOPAS substrate through repeating transfer and growth methods. Each layer of the graphene ribbon array is patterned using electron beam lithography and oxygen plasma etching. Throughout this process, gold films are placed on the graphene as source electrodes with simultaneous electron beam evaporation and lift-off techniques. Lastly, the entire device can be exfoliated from the silicon substrate.

## 3. Results and Discussion

### 3.1. Broadband Low-Dispersion Phase Gradient

To obtain a wideband low-dispersion phase gradient to achieve the broadband dispersion effect of THz waves, it is required that the reflection phase variation of the unit cell as a function of the Fermi level be approximately dispersionless over a wide band. Furthermore, the variable reflection phase through the unit cell must cover the 2π range to meet the phase accumulation requirement for generating abnormal reflection and beam-focusing phenomena at the metasurface. In the designed metasurface, graphene ribbons have the same width much smaller than the incident wavelength, but show different sheet conductivity depending on their Fermi levels. Each graphene ribbon in the unit cell works as an electric dipole, and the whole unit cell serves as a Fabry–Perot resonant cavity. When the graphene dipole resonance overlaps with the Fabry–Perot resonance, the interference between them produces the expected control for both the amplitude and phase of the reflected wave. Thereupon, such an interference effect brings a large accumulation of the phase shifts for the nearly 2π-coverage, while maintaining the high reflection amplitude of the unit cell [39].

**Figure 3:**
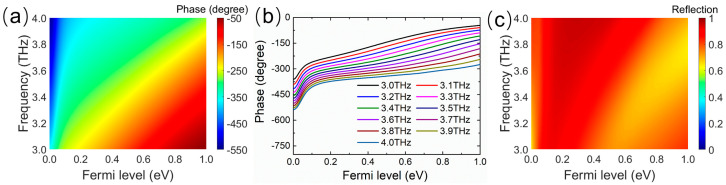
(**a**) Reflection phase map as a function of the Fermi level and frequency. (**b**) Variation in reflection phase with Fermi level at different frequency points. (**c**) Reflection amplitude map as a function of the Fermi level and frequency.

As shown in Figure 3, in the wide incidence spectra range of 3.0−4.0 THz, the Fermi level graphene in the unit cell is swept under normal incident THz excitation, and the reflection amplitude and phase spectra of the unit cell are obtained for different Fermi levels of graphene. Figure 3a depicts the reflection phase spectra of the unit cell by shifting the Fermi level from 0 eV to 1.0 eV, which reveals that the reflection phase has a large enough broadband tunability. By increasing the Fermi level, a large accumulation of the phase shifts can be achieved at any frequency point in the band from 3.0 THz to 4.0 THz. Moreover, the reflected phase as a function of the Fermi level is similar at different frequency points, suggesting the phase gradient depending on the Fermi level does not differ much with frequency, as exhibited in Figure 3b. This implies that the phase gradient of the metasurface composed of graphene ribbons with different Fermi levels will exhibit low dispersion in the operating frequency band. As shown in Figure 3c, in the map of the amplitude as a function of the Fermi level and frequency, the reflection amplitudes are above 0.8 in most regions and always exceed 0.65 in the whole area. Therefore, the desired metasurface with a low-dispersion phase gradient and high reflection efficiency can be constructed by arranging the unit cell with different Fermi levels. Because the reflected phase can be locally engineered by changing the Fermi level of each graphene ribbon, the wavefront of the reflection wave can be dynamically fully controlled in a wide band.

### 3.2. Single-Beam Dispersion Control 

**Figure 4:**
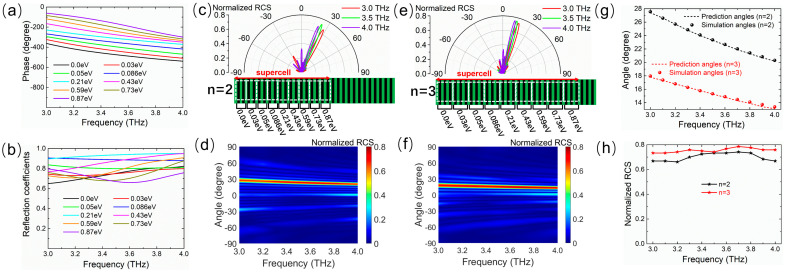
Frequency spectra of (**a**) reflection phase and (**b**) amplitude of the unit cell at nine different Fermi levels (0.0, 0.03, 0.05, 0.086, 0.21, 0.43, 0.59, 0.73, and 0.87 eV) for the normal THz wave illumination. Frequency dependencies of the abnormal reflection angle for *n* = 2 in (**c**,**d**) and *n* = 3 in (**e**,**f**), where (**c**,**e**) show the 2D far-field scattering patterns in polar coordinates for a normal incidence wave at 3.0, 3.5, and 4.0 THz, and (**d**,**f**) show the normalized RCS map as a function of the frequency and spatial angle. (**g**) Curves of simulated and predicted reflection angles and (**h**) normalized RCS at main lobe direction versus frequency for *n* = 2 and *n* = 3. The angle range of the beam reflection dispersion is tuned by changing the repetition number *n*.

Relying on the Fermi level *E*_F_, the phase can change over a range of 2π, and the desired phase gradient can be achieved by arranging the sequence of Fermi levels of graphene ribbons via the bias voltage. Then, the Fermi levels of graphene are freely chosen to construct the expected tunable metasurface with a broadband dispersion effect. As a proof-of-concept example, we first explore the abnormal reflection dispersion of a single beam on a phase gradient metasurface, which works as a tunable reflective planar prism. For this purpose, nine different Fermi levels are selected for the unit cells and arranged to create a linear discrete phase distribution with equal intervals of 2π/9 along the metasurface. Figure 4 shows the reflection phase and amplitude spectra of the unit cell corresponding to nine different Fermi levels. The phase gradient remains roughly constant in the wide band from 3.0 THz to 4.0 THz, as shown in Figure 4a, and the reflection amplitudes are above 0.65 in the whole band, as shown in Figure 4b. The abnormal reflection angle is determined by the generalized Snell’s law that is simplified as follows for the normal incidence case from the air [5]:

(5) $$\sin(\theta_{r})=\frac{c}{2\pi f}\frac{d\Phi }{dx}$$

where *θ_r_* is the angle of the reflection direction, and *c* and *f* are the speed of light in free space and the frequency of the incident wave, respectively. *d*Φ/*dx* represents the phase gradient and approaches 2π/*P* in the designed metasurface, where *P* is the geometric period of a supercell and is determined by the total number of graphene ribbons. The supercell is a periodical repetitive part with a 2π phase shift, as shown in Figure 4c. For a certain frequency, the abnormal reflection angle *θ_r_* simply depends on 2π/*P*. To control the reflection angle, the period *P* of the supercell can be changed by increasing the repetition number *n* of each pre-selected Fermi level.

Figure 4c shows the two-dimensional (2D) far-field scattering patterns in the *xoz*-plane of the metasurface for *n* = 2 with normal incidence waves at 3.0 THz, 3.5 THz, and 4.0 THz whose corresponding reflection angles are 27.4°, 23.3°, and 20.2°, respectively. Figure 4d shows the normalized RCS map as a function of frequency and spatial angle in the *xoz*-plane. It can be seen that the incident waves are mostly reflected along different abnormal angles that vary with the increase in the frequency. After changing the repetition number *n* of each Fermi level in Figure 4a, for example, *n* = 3, similar dispersion results are observed, as shown in Figure 4e,f. Nevertheless, the angle range of the main lobe direction is translated by increasing *n*, and the reflection angles change into 17.8°, 15.3°, and 13.1° at 3.0 THz, 3.5 THz, and 4.0 THz, respectively. As illustrated in Figure 4g, it is found that the reflection angle decreases approximately linearly with the blue-shifting of the frequency in the working band from 3.0 THz to 4.0 THz for both *n* = 2 and *n* = 3. The angular dispersion rates with respect to the wavelength are 0.29°/μm (*n* = 2) and 0.19°/μm (*n* = 3), and the simulated angles are in good agreement with the theoretical prediction based on the generalized Snell’s law. The relationships between the normalized RCS at the main lobe and the frequency shown in Figure 4h illustrate the fact that the reflection coefficients are at least 0.66 and 0.73 for *n* = 2 and *n* = 3, respectively. As a result, in accordance with the low-dispersion nature of the phase gradient within a wide band, the reflection angle is merely dependent on the frequency according to Equation (5), thereby bringing about the broadband angle dispersion of the abnormal reflection direction of the single beam. Moreover, the angle range of beam reflection dispersion can be dynamically tuned by varying *n*, where *n* is expediently varied by applying different bias voltage distributions instead of tailoring the geometry and size of the metasurface.

**Figure 5:**
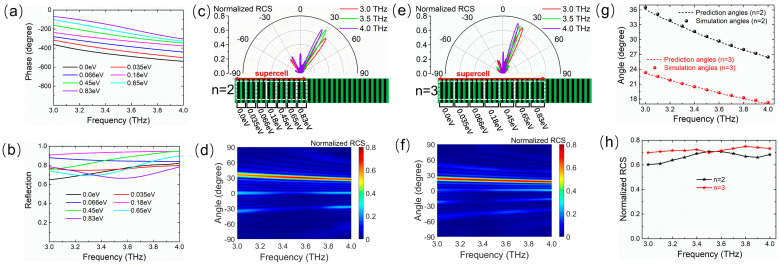
Frequency spectra of (**a**) reflection phase and (**b**) amplitude of the unit cell at seven different Fermi levels (0.0, 0.035, 0.066, 0.18, 0.45, 0.65, and 0.83 eV) for the normal THz wave illumination. Frequency dependencies of the abnormal reflection angle for *n* = 2 in (**c**,**d**) and *n* = 3 in (**e**,**f**), where (**c**,**e**) show the 2D far-field scattering patterns in polar coordinates for a normal incidence wave at 3.0, 3.5, and 4.0 THz, and (**d**,**f**) show the normalized RCS map as a function of the frequency and spatial angle. (**g**) Curves of simulated and predicted reflection angles and (**h**) normalized RCS at main lobe direction versus frequency for *n* = 2 and *n* = 3. The angle range of the beam reflection dispersion is tuned by changing the repetition number *n*.

In addition to increasing *n*, the angle dispersion range is regulated by resetting the abrupt phase interval to change the total number of graphene ribbons in a supercell. Figure 5a shows the reflection phase spectra of the unit cell corresponding to seven different Fermi levels, which can be combined to create the 2π-coverage of the phase shifts in the supercell. These seven Fermi levels also offer an abrupt phase interval of 2π/7 along the metasurface to form a low-dispersion phase gradient in the working band from 3.0 THz to 4.0 THz. Figure 5b shows the reflection amplitude spectra of the unit cell at seven Fermi levels. The amplitudes are also greater than 0.65 in the whole band. To contrast with the condition shown in Figure 4, we investigated the supercell constructed with the seven Fermi levels corresponding to *n* = 2 and *n* = 3. With the exception of the different dispersion angle ranges, similar tunable dispersion functions are shown in Figure 5c–h. When *n* = 2, the abnormal reflection angles are 36.1°, 30.5°, and 26.4° for normal incidence waves at 3.0 THz, 3.5 THz, and 4.0 THz, respectively (see Figure 5c). When *n* = 3, the abnormal reflection angles are 23.2°, 19.9°, and 17.1° at the frequencies of 3.0 THz, 3.5 THz, and 4.0 THz, respectively (see Figure 5e). In addition, it is observed that the THz wave is exclusively being anomalously redirected in the entire wavelength range for both *n* = 2 and *n* = 3 (Figure 5d,f). It is also seen that the correlation between the reflection angle and the frequency is linear negative with the angular dispersion rates of 0.39°/μm (*n* = 2) and 0.24°/μm (*n* = 3), and the simulated angles perfectly match the theoretical prediction, as shown in Figure 5g. The reflection coefficients at the main lobe are greater than 0.6 and 0.7 at any operating frequency for *n* = 2 and *n* = 3, respectively, as shown in Figure 5h. Thus, it is indicated that the angle dispersion range can be dynamically tuned by either resetting the abrupt phase interval or varying *n* and holding the broadband dispersion effect under different conditions in the frequency range from 3.0 THz to 4.0 THz.

### 3.3. Dual-Beam Dispersion Control 

**Figure 6:**
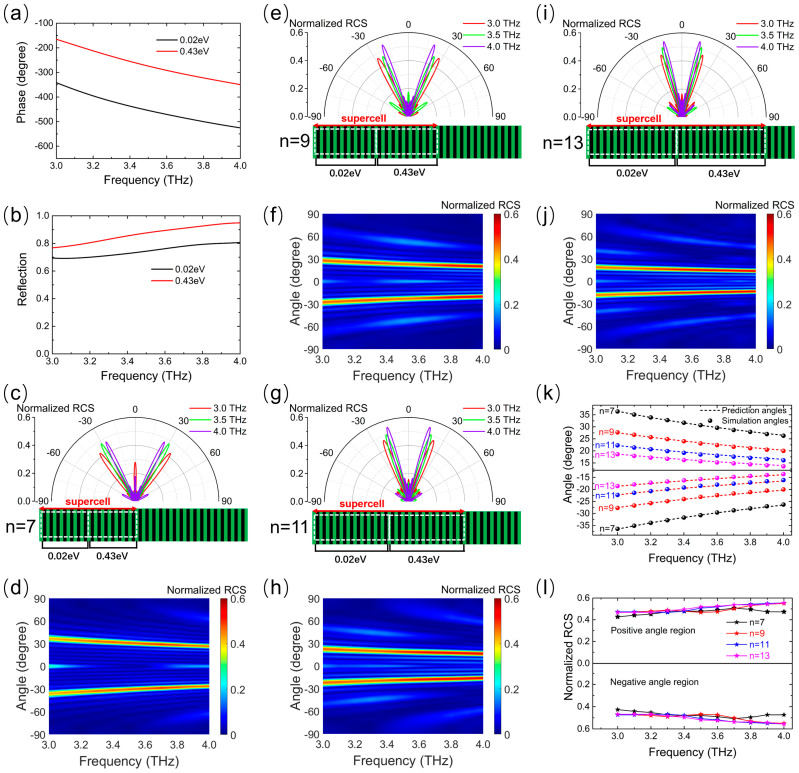
Schemes follow the same formatting. Frequency spectra of (**a**) reflection phase and (**b**) amplitude of the unit cell at two Fermi levels (0.02 and 0.43 eV) for the normal THz wave illumination. Frequency dependencies of the abnormal reflection angle for *n* = 7 in (**c**,**d**), *n* = 9 in (**e**,**f**), *n* = 11 in (**g**,**h**), and *n* = 13 in (**i**,**j**), where (**c**,**e**,**g**,**i**) show the 2D far-field scattering patterns in polar coordinates for a normal incidence wave at 3.0, 3.5, and 4.0 THz; (**d**,**f**,**h**,**j**) show the normalized RCS map as a function of the frequency and spatial angle. (**k**) Curves of simulated and predicted reflection angles and (**l**) normalized RCS at main lobe direction versus frequency for different *n* used to tune the angle range of dual-beam reflection dispersion.

To further demonstrate the tunable dispersion effect of the reflection direction on the presented metasurface, we investigated the tunable wideband dispersion properties of dual-beam abnormal reflections. As examples of verification, two different Fermi levels (0.02 eV, 0.43 eV), with identical phase differences of π (see Figure 6a) and high reflection amplitudes (see Figure 6b) in a broad band from 3.0 THz to 4.0 THz, were selected to construct the desired tunable dispersive metasurface with double abnormal reflection beams. In a similar way to that discussed before, each graphene ribbon with the same width but a different Fermi level was arranged alternately after repeating a certain number of times (*n* = 7, *n* = 9, *n* = 11, and *n* = 13) to form a low-dispersion phase gradient along the metasurface. The 2D far-field patterns and normalized RCS map for normal incidence were obtained, as depicted in Figure 6c–l. The reflected wave was restrained in the backward direction. Then, two abnormal reflection beams were produced along symmetrically oriented directions depending solely upon frequency for every *n*. As depicted in Figure 6k, the different angle ranges were achieved with different *n*, as predicted by the generalized Snell’s law, and the angular dispersion rates were 0.40°/μm, 0.31°/μm, 0.25°/μm, and 0.20°/μm for *n* = 7, *n* = 9, *n* = 11, and *n* = 13, respectively. As the frequency blue-shifts, both the positive and negative reflection angles decrease approximately linearly (see Figure 6k), but otherwise, the normalized RCS at the main lobe slightly increases (see Figure 6l) due to the reflection amplitudes of the unit cell increasing with the increase in the frequency (see Figure 6b). Hence, in the wide working band from 3.0 THz to 4.0 THz, the proposed metasurface proves the angle dispersion of the double THz reflection beam, and the angle dispersion range can be dynamically tuned by adjusting the sequence of the Fermi levels.

### 3.4. Dispersion Control of Beam Focusing

The proposed scheme is also a good candidate for achieving the broadband dispersion of beam focusing and the dynamical control of the focal length range. When the THz wave is normally incident on the metasurface along the *z*-direction, abrupt phase changes with a specific distribution at the interface are produced by unit cells, and the reflected beam is focused at a particular focal length. According to Fermat’s principle, the compensated phase distribution generated by the unit cells on the metasurface interface can be expressed as follows [53]:

(6) $$\varphi =\frac{2\pi f}{c}(\sqrt{x^{2}+F^{2}}-F)$$

where *F* is the expected focal length, *x* is the distance to the center of the metasurface, and *c* and *f* are the speed of light in free space and the frequency of the incident wave, respectively. To prove the ability of the metasurface for the dynamical dispersion control of beam focusing, we design three different sequence arrangements of the Fermi level (S1, S2, and S3) that allow the metasurface to work as a metalens with tunable dispersion ranges of the focal length. In each sequence arrangement of the Fermi level, every two adjacent unit cells with the same Fermi level are responsible for generating an abrupt phase change. The phase required for the geometric position of the metalens can be calculated using the above Equation (6). Then, the Fermi level corresponding to the phase at each position can be obtained by applying the relationship between the reflected phase and the Fermi level of graphene given in Figure 3a.

**Figure 7:**
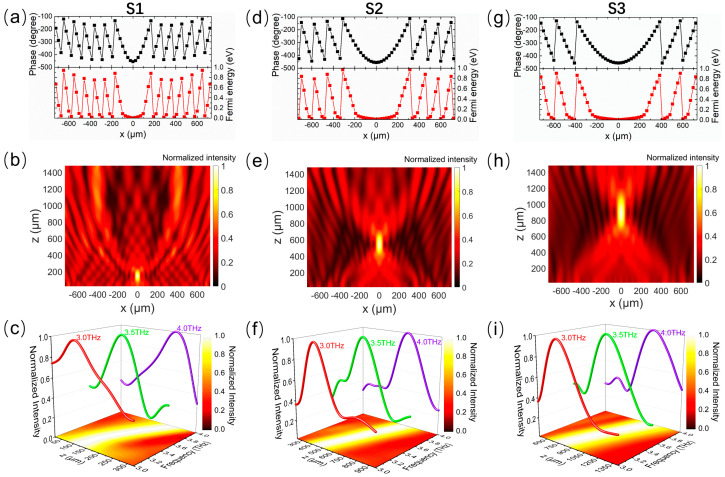
Phase profile of the metalens and reflected field intensity distribution for (**a**–**c**) S1, (**d**–**f**) S2, and (**g**–**i**) S3. (**a**,**d**,**g**) Phase distribution and the corresponding Fermi levels of graphene ribbons at different positions of the metalens along the *x*-direction. (**b**,**e**,**h**) Normalized field intensity distribution in the *xoz*-plane for normal incidence at 3.4 THz. (**c**,**f**,**i**) Normalized field intensity distribution at the center of the metalens along the *z*-axis at 3.0 THz, 3.5 THz, and 4.0 THz, and map of the normalized field intensity as a function of the frequency and position for the *z*-axis, where the peak value of field intensity corresponds to the position of the focal point on the *z*-axis.

Figure 7a shows the phase distribution and sequence arrangement of the Fermi level for S1. In this state, the metasurface is expected to function as a metalens with a focal length of 140 μm at 3.4 THz. It can be seen from the normalized electric field intensity distribution shown in Figure 7b that the electric field intensity is concentrated at 139.5 μm and the simulation results are in good agreement with the theoretical design objective. In addition, because the phase gradient formed by the different Fermi levels has low dispersion in a wide band, the metasurface becomes a planar reflective lens with the broadband dispersion effect. As shown in Figure 7c, the metalens has a good focusing effect for the THz wave with different wavelengths in the working band from 3.0 THz to 4.0 THz, and the focal length is positively correlated with the frequency. For S2 and S3, whose focal lengths are designed to be 540 μm and 900 μm, respectively, at 3.4 THz, Figure 7d–i show the phase distribution and sequence arrangement of the Fermi level, as well as the corresponding normalized field intensity in the *xoz*-plane at 3.4 THz and along the *z*-axis at different wavelengths. It is found that the focal points are at 530.5 μm and 867.5 μm for S2 and S3, respectively, as shown in Figure 7e,h, which is also almost consistent with the theoretical design objective. The focal length increases approximately linearly with the increase in the frequency but with different focal length ranges, as shown in Figure 7f,i, in the whole operating band.

**Figure 8:**
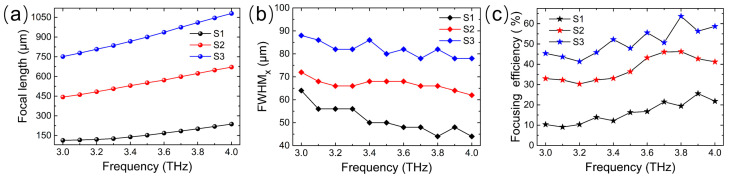
Frequency dependencies of (**a**) focal length, (**b**) full width at half maximum along the *x*-axis, and (**c**) focusing efficiency of the designed metalens corresponding to three different sequence arrangements of the Fermi level (S1, S2, and S3).

The frequency dependencies of the focal length for S1, S2, and S3 are contrasted in Figure 8a, which reveals that the metasurface achieves broadband dispersion of the beam focusing, and the variation range of the focal length is dynamically tuned by changing the sequence arrangement of the Fermi level. The dispersion ranges of the focal length are 113.5–237.5 μm, 443.5–671.5 μm, and 751.5–1079.5 μm for S1, S2, and S3, respectively, and the corresponding focal length dispersion rates with respect to the wavelength are 4.96, 9.12, and 13.12, respectively. This means that the focal length of the metalens can be freely regulated from about one wavelength to ten wavelengths through the dispersion effect and sequence arrangement of the Fermi level. To quantitatively analyze the focusing effect, the full width at half maximum (FWHM) of the normalized electric field intensity in the focal plane along the *x*-direction is extracted, as shown in Figure 8b. It is observed that the greater the focal length is at the same frequency, the greater the FWHM is. The corresponding values are around 50 μm, 70 μm, and 85 μm for S1, S2, and S3, respectively. Based on the electric field intensity and FWHM in the focal plane, the focusing efficiency is also calculated with the following formula [53]:

(7) $$\eta_{s}=\frac{{\left|E_{FWHM\times 3}\right|}^{2}}{{\left|E_{total}\right|}^{2}}\times 100\%$$

where E*_FWHM_*_×3_ is the electric field energy within the range of the triple FWHM in the focal plane and E*_total_* is the electric field energy of the incident wave through the focal plane. The results in Figure 8c represent the frequency dependencies of the focusing efficiency of the metalens for S1, S2, and S3. Because the reflection amplitude of the unit cell increases slightly with the increase in the frequency, the focusing efficiency shows an overall upward trend as the frequency increases. Additionally, with the focal length increasing at the same frequency, the efficiency increases significantly. This is due to the fact that the focal spot becomes larger as the focal length increases, leading to an increase in FWHM, and the efficiency calculated according to Equation (7) also increases. As a result, the metasurface can achieve the dispersion control of beam focusing, including broadband dispersion of the focal length and dynamical tuning of the focal length range in the working band from 3.0 THz to 4.0 THz.

### 3.5. The Performances under Oblique Incidence

**Figure 9:**
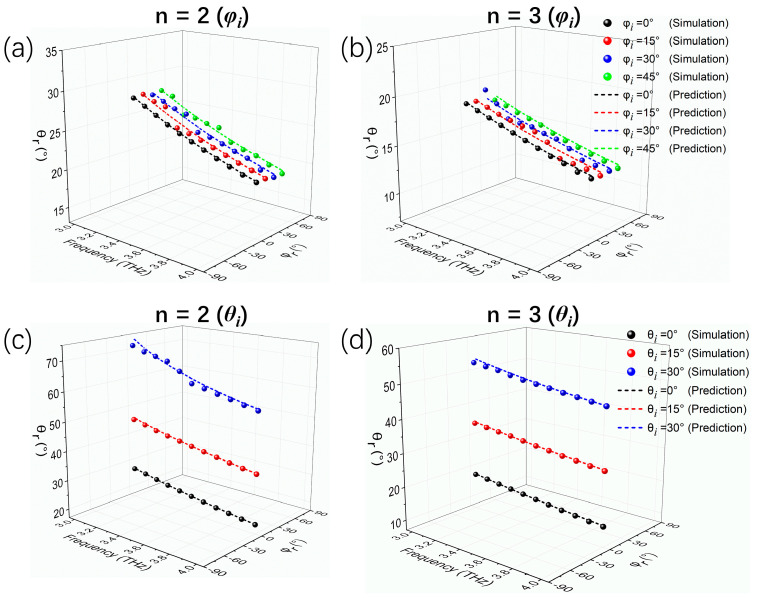
Curves of simulated and predicted anomalous reflection angles versus frequency for *n* = 2 and *n* = 3 under different incident directions in (**a**,**b**) *yoz*-plane and (**c**,**d**) *xoz*-plane. The angle range of the beam reflection dispersion can also be regulated by changing *n*.

In addition, we have also investigated the dispersion manipulation performances of the graphene-enabled tunable metasurface under oblique incidence. As a proof-of-concept example, here we only consider that the abrupt phase shift along the metasurface is 2π/9, corresponding to *n* = 2 and *n* = 3. Figure 9 displays the simulation and prediction results for incidence in the *yoz*-plane with different angle *φ_i_* values and in the *xoz*-plane with different angle *θ_i_* values. For incident angle *φ_i_*, the device achieves tunable broadband dispersion of reflection angle *θ_r_* up to 45° of either *n* = 2 or *n* = 3 in the working band, as shown in Figure 9a,b. Here, the changes in reflection angle *φ_r_* (*φ_r_* = *φ_i_*) result from the specular reflection caused by oblique incidence in the *yoz*-plane. Under oblique incidence with the angle *θ_i_*, the broadband dispersion characteristics of the reflection angle *θ_r_* are well maintained up to 30° for both *n* = 2 and *n* = 3, and the anomalous reflection angles are in good agreement with the theoretical predictions, as shown in Figure 9c,d. When the incidence angle *θ_i_* is larger, the reflection angle *θ_r_* will exceed 90° according to the generalized Snell’s law [5], and the incident wave might be converted to a surface wave. Overall, the results imply that the broadband dispersion characteristics are insensitive to the wide angle of oblique incidence within the frequency range from 3.0 to 4.0 THz. In addition, the angle range of the beam reflection dispersion can also be dynamically regulated by changing *n* under different oblique incidence angles.

## 4. Conclusions

In summary, we have presented a graphene-enabled tunable metasurface for the dynamic control of the dispersion effect of a wideband THz wave. The unit cell exhibited tunable phase spectra on the Fermi level of graphene, introducing various broadband low-dispersion phase gradients along the metasurface. Full-wave simulation experiments demonstrated that wideband dispersion and dynamical tuning for both beam reflection and beam focusing were achieved by the metasurface in the broad operating band from 3.0 THz to 4.0 THz. The abnormal reflection angle and focal length were merely dependent on the frequency over a wide THz band for the low-dispersion nature of the phase gradient, and the focal length range and reflection angle range based on dispersion could be expediently varied on a large scale by engineering the Fermi level distribution of graphene. The insensitivity of the metasurface under oblique incidence has been revealed through simulation experiments, indicating good robustness in practical application. Table 1 compares the tunable phase gradient metasurfaces proposed in this work with existing devices.

From the comparison, it can be found that the presented device has broadband dispersion characteristics, and the dispersion range can be dynamically regulated as demanded. Thanks to the combination of broadband dispersion and the Fermi level, the variable spatial range of beam steering and beam focusing is further extended, enhancing the flexibility of the device in practical applications. The proposed scheme paves the way for the next potential applications of metasurfaces for advanced light control, such as optics, imaging, and wireless communication.

## Figures and Tables

**Table 1:** Comparison of the tunable phase gradient metasurfaces.

Ref.	Frequency	Dispersion Control	Tunable Mechanism	Function
[42]	35.29 THz	No	Fermi level	Beam steering/beam focusing
[43]	55 GHz	No	Sheet resistance	Multiple-beam steering
[44]	1.86 THz	No	Fermi level	Beam focusing
[45]	6.15 THz	No	Fermi level	Absorption/multi-focus/self-healing
[46]	1.14 THz	No	Fermi level	Beam forming/beam steering
This work	3.0–4.0 THz	Yes	Fermi + frequency	Beam steering/beam focusing/regulatable dispersion range

## Data Availability

Data are available on request from the authors.
